# Neuropsychiatric symptoms in frontotemporal dementia: a case report

**DOI:** 10.1192/j.eurpsy.2024.1329

**Published:** 2024-08-27

**Authors:** A. Izquierdo De La Puente, P. del Sol Calderón, R. Fernandez Fernandez

**Affiliations:** ^1^Psychiatry, Hospital Universitario Puerta de Hierro de Majadahonda, Majadahonda; ^2^Psychiatry, Hospital Universitario Infanta Cristina, Parla, Spain

## Abstract

**Introduction:**

We present the case of a 70-year-old man who, after presenting atypical depressive symptoms, was diagnosed with incipient frontotemporal dementia.

**Objectives:**

Through the presentation of the case, a brief review is made of the affective prodromes of frontotemporal dementia

**Methods:**

The patient, who had no personal history of interest, suddenly began to present depressive symptoms consisting of marked irritability, dysphoric mood, anxious semiology with a subjective feeling of anguish, maintenance insomnia and a feeling of lack of self-control, with a tendency towards verbal heteroaggressiveness. The patient reported all these symptoms with great suffering.

After one year of treatment with venlafaxine 300g DMD and quetiapine 400g DMD, with one admission to the short-stay inpatient unit for self-harm threats, the patient had not experienced any improvement. In addition, during this year, the patient’s family began to observe small memory lapses that affected his daily functioning, making the patient progressively more dependent.

**Results:**

In view of this clinical picture, it was decided to request an MRI and a brain PET scan, where deficits in the frontal and temporal regions were observed, and a diagnosis of incipient frontotemporal dementia was made.

**Conclusions:**

Frontotemporal dementia is the third most common dementia in people over 65 years of age. About half of the patients debut with psychiatric symptoms, one of them being depressive symptoms. Treatment is focused on the use of psychotropic drugs with the aim of symptom management. Olanzapine or aripiprazole are effective for psychotic symptoms or acute agitation. For more subacute conditions, SSRIs or trazodone are recommended. The iACOs are not recommended, because they are ineffective and worsen neuropsychiatric symptoms.

**Disclosure of Interest:**

None Declared

